# Genetic and Morphometric Divergence of an Invasive Bird: The Introduced House Sparrow (*Passer domesticus*) in Brazil

**DOI:** 10.1371/journal.pone.0053332

**Published:** 2012-12-28

**Authors:** Marcos R. Lima, Regina H. F. Macedo, Thaís L. F. Martins, Aaron W. Schrey, Lynn B. Martin, Staffan Bensch

**Affiliations:** 1 Departamento de Ecologia – IB, Pós-Graduação em Ecologia, Universidade de Brasília, Brasília, Brazil; 2 Departamento de Zoologia – IB, Universidade de Brasília, Brasília, Brazil; 3 Centre for Ecology and Conservation, University of Exeter, Cornwall Campus, Tremough, Penryn, Cornwall, United Kingdom; 4 Department of Integrative Biology, University of South Florida, Tampa, Florida, United States of America; 5 Department of Biology, Lund University, Lund, Sweden; CNRS, Université de Bourgogne, France

## Abstract

Introduced species are interesting systems for the study of contemporary evolution in new environments because of their spatial and temporal scales. For this study we had three aims: (i) to determine how genetic diversity and genetic differentiation of introduced populations of the house sparrow (*Passer domesticus*) in Brazil varies with range expansion, (ii) to determine how genetic diversity and differentiation in Brazil compares to ancestral European populations; and (iii) to determine whether selection or genetic drift has been more influential on phenotypic divergence. We used six microsatellite markers to genotype six populations from Brazil and four populations from Europe. We found slightly reduced levels of genetic diversity in Brazilian compared to native European populations. However, among introduced populations of Brazil, we found no association between genetic diversity and time since introduction. Moreover, overall genetic differentiation among introduced populations was low indicating that the expansion took place from large populations in which genetic drift effects would likely have been weak. We found significant phenotypic divergence among sites in Brazil. Given the absence of a spatial genetic pattern, divergent selection and not genetic drift seems to be the main force behind most of the phenotypic divergence encountered. Unravelling whether microevolution (e.g., allele frequency change), phenotypic plasticity, or both mediated phenotypic divergence is challenging and will require experimental work (e.g., common garden experiments or breeding programs).

## Introduction

Species invasions provide an opportunity to examine fundamental questions in ecology and evolutionary biology, such as changes in geographical ranges, reproductive isolation and adaptation to novel environments, due to the large spatial and temporal scale of these “unplanned experiments” [1]. Bird introductions provide exceptionally good study opportunities because excellent historical records are often available, such as date of introduction, number of individuals released, number of introductions and locality where individuals were released [2], [3]. These data allow us to study evolution of species in new environments and over ecological time scales. More specifically, such instances generate data that can be used to examine how genetic diversity relates to range expansion [4]–[6] and the effects that selection and genetic drift may have on population divergence [7]–[9]. Most studies of non-native species have focused on *ecological* aspects of invasions, whereas *evolutionary* aspects have been less studied [10], [11]. Therefore, incorporating the change in genetic and phenotypic properties due to evolution in the introduced environments may help to predict establishment success and impacts of non-native species [12]. For example, many introduced species only become invasive after a lag phase, which could be associated with the time that is necessary for evolutionary adjustments to take place [13], [14].

In general, the number of released individuals and introduction events (propagule pressure) are associated with the success of establishment and spread of invasive species [15]. These relationships are thought to exist because population size is tightly linked to demographic, environmental and genetic stochasticity [12], [15], [16]. Indeed, introduced populations tend to lose significant genetic diversity (i.e., allelic richness and/or heterozygosity), because of founder events [6]. However, many invasive species show only modest reductions in genetic diversity [17], which could be due to large propagule pressure, especially if propagules originated from different areas in the native range [6], [16], [18]. It is possible that for a significant decrease in genetic diversity to occur after an introduction event, a multiple step-wise colonization process (i.e., sequential founder events) may be necessary [19], or in the case of multiple introductions, that gene flow in the introduced range be constrained [18]. In introduced birds, there is evidence for both loss of genetic diversity [20]–[25] and no change in genetic variability [7], [24], [26], [27]. However, the loss of genetic variation in introduced bird populations is associated with low propagule pressure and/or slow population growth rate after introduction [23], [28].

Studying genetic diversity and population structure across the range of a broadly distributed invasive species can help reveal the mechanisms that generate differentiation, as well as provide insight into colonization dynamics [4], [12]. For instance, the expansion of an invasive species can be a contiguous or non-contiguous process and can be accompanied by a large increase in the number of individuals, which together with the mode of dispersal will affect population genetic structure [5], [29], [30]. If dispersal (i.e., gene flow) between close populations is more frequent than between populations further apart (moderate dispersal), an isolation by distance pattern should be expected. Conversely, no pattern of geographic genetic differentiation may occur in the introduced range if gene flow within the introduced range is strong relative to genetic drift, especially if the source propagule was genetically homogeneous prior to the introduction (e.g. low propagule pressure). However, if gene flow is low among the expanding population fragments, then genetic drift will increase genetic differentiation and this process will be relatively independent of geographic distance. Over time, gene flow between adjacent population will form a pattern of isolation by distance but such an equilibrium between drift and gene flow might not develop during the limited time frame (e.g. a couple of hundred years) of most invasive species (see Figure 1 in [5], [31]).

**Figure 1:**
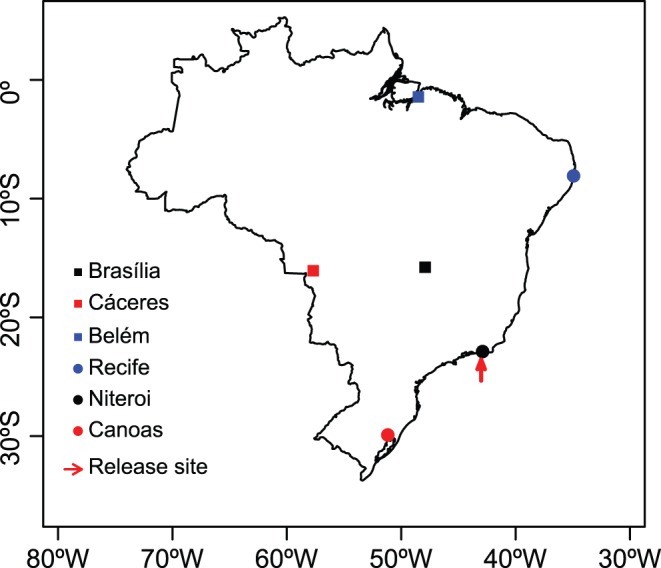
Map of Brazil showing the different house sparrow populations sampled and the location of the release site.

To better understand rapid evolution and how invasive species adjust to novel environments, population genetic studies should be combined with analyses of spatial phenotypic differentiation [9]. For example, some invasive species have the capacity to expand or shift their niches [13], [32], which is probably a response to novel selective pressures. If this is generally the case, then a response in quantitative traits can be expected if there is enough genetic and/or quantitative variation for selection to act upon [17]. In support, there are examples of introduced species that exhibit clinal patterns in morphology [33], [34], as might be expected with the above scenario. However, untangling whether selection or genetic drift is responsible for phenotypic divergence is a complex task, and requires the initial step of comparing spatial genetic differentiation (*F_ST_*) with spatial phenotypic differentiation (*P_ST_*). If *P_ST_* is significantly larger or smaller than *F_ST_*, then it is possible that the geographic variation in phenotypic traits were more likely shaped by selection rather than governed by genetic drift [35]–[37]. *P_ST_* is a rough estimate of *Q_ST_*, the latter measures variation in quantitative traits by partitioning the variance related to additive genes between and within populations [38]. However, attaining the necessary information for calculating *Q_ST_* can be challenging because it requires the rearing of several populations in common garden conditions. Therefore, phenotypic measures have been used as a surrogate, but one should be cautious to the possible caveats associated with the use of *P_ST_* [39].

In this study we used the house sparrow (*Passer domesticus*) to address three main questions. First, we compared genetic diversity and population structure between populations in the introduced range in Brazil and the native range in Europe. We expected populations from Brazil to be less diverse than the European populations. Second, we analyzed genetic differentiation among populations in Brazil to understand how the expansion process in Brazil occurred. For example, if house sparrows in Brazil underwent sequential founder events during its expansion, one would expect a pattern of isolation by distance and populations in the expansion front to present reduced genetic diversity. Our third goal was to compare spatial phenotypic differentiation (*P_ST_*) with genetic differentiation (*F_ST_*). This method would allow us to evaluate whether phenotypic differences between populations were shaped by selection or genetic drift. In principle, if *P_ST_* equals *F_ST_*, differentiation of morphological traits (assumed to be governed by additive genetic variation) is probably the result of genetic drift. However, if *P_ST_* is larger than *F_ST_* it means that quantitative traits have diversified more than neutral genetic loci, which could be evidence of directional selection. Alternatively, if *P_ST_* is significantly smaller than *F_ST_*, quantitative traits probably diversified less than neutral genetic loci, suggesting that these traits have been under the influence of stabilizing selection [34]–[36].

We chose the house sparrow as our model because of its remarkably broad distribution, predominantly determined by human introductions [3], [40]. This distribution allows for multiple study replicates because genetic data from the introduced ranges of North America, Kenya, Australia and New Zealand [24], [26] already exist. Data on morphological divergence also exists from North America [34], South America [41] and New Zealand [42].

## Materials and Methods

### Ethical Statement

This study was carried out in accordance with current laws of all countries where the study was performed and followed the recommendations of the Guidelines to the Use of Wild Birds in Research (Fair, J. E. Paul, and J. Jones, Eds 2010. Washigton, D.C.: Ornithological Council). In Brazil approval by an ethical committee of the university is required only for captive animals used in experiments. All other types of work with animals are regulated by IBAMA - Instituto Brasileiro do Meio Ambiente e dos Recursos Naturais Renováveis, which substitutes the ethic committee in approving and evaluating all aspects of projects that involve capture and handling of animals, taking morphological measurements, blood sampling and other procedures. Permits were obtained from IBAMA (179/2006-CGFAU; 123221 and 12322-2) for the purpose of this study and MR Lima’s PhD Project and house sparrows were captured [with mist nets] at university campi and private homes [in the cities of Brasília, Belém, Cáceres, Canoas, Niteroi and Recife], with appropriate permissions of institutions and land owners. After being measured and sampled for blood and feathers, all birds were immediately released. Field methods were carried out so that handling time and potential suffering of animals were minimized. House sparrows are considered exotic birds in Brazil, and in the IUCN Red List the species has a Least Concern category. Data on European populations were obtained from a previous study [24] with the permission of A Marzal and P Zehtindjiev. Blood samples from European house sparrows were obtained [with permission from A Marzal and P Zehtindjiev authors of] a previous PLoS ONE study [43], which was approved by the Swedish Ethical Committee on Animal Experiment (reference M64-05).

### Sampled Populations

Two hundred house sparrows were released in 1905 in Rio de Janeiro, Brazil [44], and subsequent translocations and natural expansions of established populations have spread this species widely in Brazil, reaching the edges of the Brazilian Amazon in the city of Belém by 1978 [44]–[49]. Six populations from Brazil were sampled and 15 individuals from each were genetically screened (Table 1 and Figure 1). Data for four populations from Europe were obtained from a previous study (Table 1; see Figure 1 and Table 1 in [24]). Data on the year that house sparrows arrived in the different sampled locations in Brazil were obtained from the literature [44]–[49]. It was not possible to sample house sparrows from Rio de Janeiro where they were initially released [47], but we sampled house sparrows from Niteroi, which is 10 km from Rio de Janeiro. House sparrows in Brazil were caught using mist nets and blood was obtained from the brachial vein and conserved in 99% ethanol until DNA extraction.

**Table 1:** Sampled locations of Brazil (introduced) and Europe (native) with number of individuals genotyped (N) and captured (in parentheses) for which we have morphometric data, latitude and longitude in degrees, year that house sparrow arrived in the sampled location, mean number of alleles per loci (Na), allelic richness (Ar), private allelic richness (Par), observed heterozygosity (Ho), unbiased expected heterozygosity (UHe) and measure of departure form Hardy-Weinberg proportions (*F_IS_*).

Locality	N	Long	Lat	Arrival Year	Na	Ar	Ar3^1^	Par	Par3^1^	Ho	UHe	*F_IS_*
Introduced												
Brasília	15 (20)	47° 53' W	15° 47' S	1957	12.00	11.65	6.98	1.63	0.00	0.81	0.89	0.08
Cáceres	15 (31)	57° 41' W	16° 05' S	1998	11.17	10.85	7.75	0.84	0.00	0.77	0.79	0.03
Belém	15 (32)	48° 29' W	01° 27' S	1978	11.83	11.46	7.69	1.80	0.11	0.80	0.83	0.05
Recife	15 (27)	34° 55' W	08° 05' S	1963	11.33	11.23	7.55	0.93	0.33	0.71	0.84	0.16
Niterói^5^	15 (22)	43° 08' W	22° 54' S	1905	12.00	11.65	6.92	1.64	0.27	0.84	0.84	−0.01
Canoas	15 (22)	51° 11' W	29° 55' S	1925	10.50	10.16	5.94	0.98	0.00	0.70	0.76	0.08
Mean ± sd	–	–	–	–	11.47±0.59	**11.17±0.57**	**7.13±0.69**	**1.30±0.43**	**0.11±0.28**	0.77±0.06	**0.82±0.04**	0.07±0.06
Native												
Sweden	15	13° E	55° N	NA	11.50	8.88	8.00	2.91	0.77	0.75	0.89	0.17
Bulgaria	11	26° E	44° N	NA	10.50	8.92	8.77	2.37	0.65	0.83	0.89	0.08
Italy	25	14° E	41° N	NA	17.67	10.02	8.45	3.41	1.3	0.9.	0.91	0.02
Spain	21	06° W	39° N	NA	18.00	10.48	9.64	3.64	1.83	0.81	0.93	0.13
Mean ± sd	–	–	–	–	14.42±3.97	**9.57±0.80**	**8.57±0.69**	**3.08±0.56**	**1.14±0.54**	0.82±0.06	**0.91±0.01**	0.09±0.06

Mean values in bold are significantly different (p<0.05).

1 – Using only the three matching loci.

### Laboratory Procedures

Genomic DNA was extracted from blood samples using a standard protocol with overnight digestion with proteinase K and subsequent phenol-chloroform extraction and alcohol precipitation [50]. Individuals were genotyped using six microsatellite loci (Pdoµ1, Pdoµ3, Pdoµ4, Pdoµ6, Pdo8 and Pdo9; [51]–[53]), all of which were developed for house sparrows. Polymerase chain reaction (PCR) was performed in 10 µl reactions that contained 10 ng of template DNA, 5 µl of Qiagen multiplex master mix (contains pre-optimized concentrations of HotStarTaq DNA polymerase and MgCl_2_ plus dNTPs and a PCR buffer especially developed for multiplex PCR), 1 pmol of each primer (forward primers were labelled with either 6-Fam or HEX) made up to 10 µl with ddH_2_0. For PCR conditions see Information S1. We performed separate PCRs for the six loci.

PCR products of Pdoµ1, Pdoµ6 and Pdo8 were multiplexed and diluted 1∶100, while Pdoµ3, Pdoµ4 and Pdo9 were multiplexed and diluted 1∶50. These multiplex combinations were chosen so that products had different dye labels and differed in range sizes. Labelled size standard MM1000 was mixed with multiplexed PCR products and electrophoresis was conducted in a capillary ABI3730XL sequencer (Applied Biosystems). Resulting data were analysed with GeneMapper 3.0 (Applied Biosystems) for fragment size determination.

Genotyping of house sparrow populations from Europe was done in ABI 377 (Applied Biosystems; see Schrey *et al.* [24] for details), which does not use a capillary electrophoresis system. Therefore, 10 individuals sampled by Schrey *et al.* [24] were genotyped together with individuals from Brazil to check for consistent allele scoring. For Pdoµ1 and Pdo9, we obtained a perfect match, whereas for Pdoµ3 there was a 2 bp difference among the 10 individuals. Thus, we added 2 bp to the house sparrow sampled in Brazil to attain a perfect match with this locus as well. The remaining 3 loci did not match between studies, however, for Pdoµ4 and Pdoµ6, we obtained a perfect match for homozygosity and heterozygosity (i.e., individuals that were homozygous and heterozygous in Schrey *et al.* [24] were also homozygous and heterozygous in our analysis). We did not get a perfect match for Pdo8, thus, for the analyses below, genetic comparisons were done with and without the presence of Pdo8. Because results did not change when we excluded Pdo8 from the analysis, Pdo8 was maintained in the analysis. Unless otherwise stated, we only show results with Pdo8.

### Genetic Diversity

For each of the six microsatellite loci and for each population, we tested for linkage disequilibrium (LE) and Hardy-Weinberg equilibrium (HWE) using FSTAT version 2.9.3 [54]. We observed no significant deviations from LE or HWE after correcting for multiple testing, except Pdoµ1 in Recife, Pdoµ6 in Spain and Pdoµ4 in Brasília and Italy that presented statistically significant heterozygote deficiency. We used Micro-Checker [55] to check for null alleles, large allele drop outs and stuttering. Indeed, Pdoµ1 in Recife had a high presence of null alleles (18%), as did Pdoµ6 in Spain (11%) and Pdoµ4 in Brasília (15%) and Italy (7%). However, when we pooled the data for analyses we saw no indication of true deviation from HWE (Table 2). Because none of the loci consistently deviated from HWE or presented null alleles, it is likely that for the significant cases above, sampling error or infrequent cases of allelic dropout may have occurred. Moreover, at least for the populations from Brazil, homozygote excess can be expected due to a founder effect.

**Table 2:** Polymorphic microsatellite loci used in genotyping house sparrow populations.

Loci	Na	N	Ho	He
Pdoµ1	20	160	0.794	0.871
Pdoµ3	16	162	0.926	0.905
Pdoµ4	126	153	0.817	0.974
Pdoµ6	83	156	0.891	0.964
Pdo8	29	159	0.563	0.650
Pdo9	25	159	0.783	0.825

For each locus we list the number of alleles (Na), number of individuals types (N), observed heterozygosity (Ho) and expected heterozygosity.

To compare genetic diversity between the native range (Europe) and the introduced range (Brazil), we calculated allelic richness (Ar) and private allelic richness (Par) for each population using HP-Rare [56] using all six loci, as well as just the three matching loci. When six loci were used, these calculations were done separately for Brazilian and European populations. In the case of the Brazilian populations these estimates were calculated using a rarefication procedure with a minimum number of 28 alleles (smallest sample size = 14 individuals), for each locus in each populations, while for European populations a minimum number of 16 alleles (smallest sample size = 8 individuals) was used. For the three loci comparisons a minimum number of 16 alleles was used. Observed heterozygosity (Ho) and unbiased expected heterozygosity (UHe) were calculated using GenAlEx version 6.1 [57] and we used FSTAT version 2.9.3 [54] to calculate number of alleles (Na) and *F_IS_*. We used non-parametric tests (Wilcox tests) to test for any differences in the genetic diversity estimators of the introduced and native house sparrow populations. We also compared these genetic diversity estimators with data from the literature of other house sparrow populations for studies that has used similar procedures to calculate these estimators [24], [58], [59].

To test whether house sparrow populations in Brazil had experienced a recent bottleneck, as might be expected if the expansion process occurred via sequential founder effects or because of very small initial population size at the time of release, we used BOTTLENECK version 1.2.02 [60]. The expected heterozygosity in BOTTLENECK was calculated under the Two-Phase Model (TPM) allowing for 95% single-step mutations and 5% multiple step mutations with a 12% variance for the multiple steps as recommended [61]. Significance of mismatch between expected and observed heterozygosity was inferred using the Wilcox test [60]. In addition, to test whether populations at the edge of expansion underwent sequential founder events, we subtracted the arrival year from 2012 (time since colonization) and used a Pearson correlation to test if there was a positive correlation between genetic diversity and time.

### Population Structure

Genetic differentiation among the introduced populations of Brazil was determined by *F_ST_* values, which were estimated according to Weir and Cockerham [62] as implemented in FSTAT version 2.9.3 [54]. *F_ST_* was estimated globally and between all pairs of introduced populations. Significance of global *F_ST_* was evaluated by permutation of genotypes among samples and calculating 95% Confidence Intervals (C.I.) by bootstrapping over loci (number of permutations was set at 1000). Pairwise *F_ST_* was tested to determine whether it was significantly different from zero by randomizing the genotypes, and a Bonferroni correction was used to control for type-I errors. We also calculated *D_est_* defined by Jost [63] because of the recent debates regarding *F_ST_* calculations when using highly polymorphic markers such as microsatellites. *D_est_* varies from zero, when there is no genetic differentiation between populations, to one when populations are completely differentiated, and was calculated using the web-based resource SMOGD [64] with 1000 bootstrap replicates and the harmonic mean of *D_est_* across loci. Moreover, we also used *R_ST_* [65] to infer population structure for populations from Brazil as implemented in R CALC [66]. This estimator is an analogue of *F_ST_*, however, it uses variance in allele size (number of repeat units) between populations, because mutations in microsatellites involve the addition or subtraction of a small number of repeat units. We used *R_ST_* because it is less sensitive to rare alleles than *F_ST_*.

We tested for isolation by distance, which is the correlation between geographical distance (using log transformation) and the degree of genetic differentiation, using a Mantel test in Arlequin version 3.5.1.2. [67] for *F_ST_*, while for *D_es_*_t_ and *R_ST_* we used the library “vegan” [68] in R 2.14.0. We also calculated global and pairwise *F_ST_* and *D_est_* (as above) for the European populations. These calculations were repeated separately for the Brazilian and European populations because only three loci matched between the studies.

### Phenotypic Data

Left tarsus, beak height, beak width and beak length of Brazilian sparrows were measured with a digital calliper (0.01 mm) and left wing, tail and body length were measured with a ruler (0.1 cm). Additionally, 770 feathers were plucked randomly from dorsal and breast areas (field procedures were conducted by MRL; samples sizes in Table 1). There is no data on breeding period for house sparrow in Brazil, but all males had black beaks, which is indicative of breeding [40], and we only found six individuals in active molt of remiges. Five feathers from each body region per individual were overlaid and taped to a black velvet substrate and feather colouration was measured using an Ocean Optics USB4000 spectrometer and a pulsed xenon light source (Ocean Optics PX-2; 220–800 nm range). All reflectance measurements were taken in relation to a WS-1SS white standard (Ocean optics, Dunedin, FL) and to the black velvet substrate (i.e., dark reference). We used a bifurcated fiber-optic measurement probe, which was maintained perpendicular to the feather surface at a fixed distance of 5 mm fixed to a probe block to eliminate external ambient light.

Spectrometric measurements were conducted with SpectraSuite software (Ocean optics) and three measurements, which consisted of 50 sequential spectra each, were taken from each sample at three random points by lifting the black block that contained the probe to ensure that a different part of the feather was being measured each time. Individual color was characterized by averaging the three spectra, which were interpolated to a step of 1 nm between 300 and 700 nm. We calculated brightness as the area under the spectra curve (i.e., value of zero meaning black and value of 100 meaning white) and UV-Chroma as the proportion of UV reflectance between 300 and 400 nm.

Phenotypic divergence (*P_ST_*) was used to infer the role of genetic drift and natural selection on the different morphological traits of house sparrow populations of Brazil by comparing it with *F_ST_*. *P_ST_* is similar to the *Q_ST_* index, which measures quantitative trait differentiation, however, *P_ST_* is influenced by environmental, non-additive genetic effects and by the interaction between the environment and genotype (see Merilä and Crnokrak [36]). Therefore, the use of *P_ST_* as an approximation of *Q_ST_* is usually not recommended [39]. However, to calculate *Q_ST_* it is necessary to estimate the additive genetic variances, information that is obtained typically by rearing individuals from different populations in a common environment, which for several reasons, especially for vertebrate species, is not always feasible. In the case of this study, the use of *P_ST_* can be justified because *Q_ST_* estimates are not available for our study populations and obtaining *Q_ST_* would be very challenging (rearing of several house sparrow populations in common-garden conditions). Further, the morphometric traits being considered in this study are known to have substantial additive genetic basis [36], [69]. Additionally, a *P_ST_*−*F_ST_* comparison can provide initial insights into the evolutionary process that has occurred during the expansion of the house sparrow in Brazil before further inquiries can be made. *P_ST_* was estimated as:


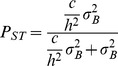


where 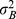 is the phenotypic variance between populations, 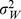 is the phenotypic variance within populations, and *h^2^* is the heritability (the proportion of the phenotypic variance attributed to additive genetic effects). The scalar *c* represents the proportion of the total variance that is claimed to occur because of additive genetic effects across the populations. If parameters *c* and *h^2^* are known for the populations being studied, then *P_ST_* equals *Q_ST_* [70]. However, estimation of *c* in the wild is very challenging and *h^2^* is population specific [39]. Because the *c/h^2^* ratio is critical to how well *P_ST_* approximates *Q_ST_*, one can use a sensitivity analysis, which varies this ratio, to infer the robustness of the approximation of *Q_ST_* by *P_ST_* [70]. According to this analysis, a null assumption would be to consider *c/h^2^* = 1 (i.e., *c* = *h^2^*), that is the proportion of phenotypic variance due to additive genetic effects is the same for both within and between population variance. If *P_ST_* exceeds *F_ST_* at this point it will also do so at any point where *c*>*h^2^* [70]. More important, however, is to evaluate if *P_ST_* exceeds *F_ST_* when *c*<*h^2^* (i.e., *c/h^2^*<1). The reason is that natural populations are probably under genotype-environmental interactions and/or divergent environmental effects and a low value of *c/h^2^* assumes a larger role of environmental effects in driving between population variance than within population variance (i.e, *c*<*h^2^*). Therefore, the lower the critical *c/h^2^* ratio is (*c/h^2^*<1) when *P_ST_* exceeds *F_ST_*, the more likely it is that the trait is being shaped by selection [70]. Therefore, if there is evidence of between population variance deriving from additive genetic effects, even in a scenario where environmental factors have a stronger role in determining phenotypic variation, then phenotypic divergence will be the result of selection, as long as the trait is heritable [71].

Variance components for estimating *P_ST_* were obtained using analysis of variance where body length was entered as a covariate. *P_ST_* 95% C.I. were calculated, by considering *P_ST_* to be normally distributed and using critical values of t, to test whether they overlapped with global *F_ST_* value ±95% C.I. and thus whether *P_ST_* values were different from *F_ST_*. The critical *c/h^2^* ratio was obtained by graphically exploring *P_ST_* and its 95% C.I. as a function of *c/h^2^* and by looking at the approximate value of *c/h^2^* where the lower 95% C.I. of *P_ST_* meets the upper 95% C.I. of *F_ST_* [70]. For example, a critical *c/h^2^*<0.1 means that in order for genetic drift to explain phenotypic divergence, the required additive genetic effect across populations would need to be less than 10% of the additive genetic effect within population. Therefore, this would be a very robust inference that the traits are under selection and not genetic drift [70].

We also conducted multivariate analysis of variance (MANOVA) to test if populationś centroids of trait means were significantly different from each other. MANOVA assumptions were checked before analysis, and three males and two females were not included in the analysis because they were multivariate outliers, which was checked with the R package “mvoutlier” [72].

## Results

### Impact of Introduction on Genetic Diversity

**Figure 2:**
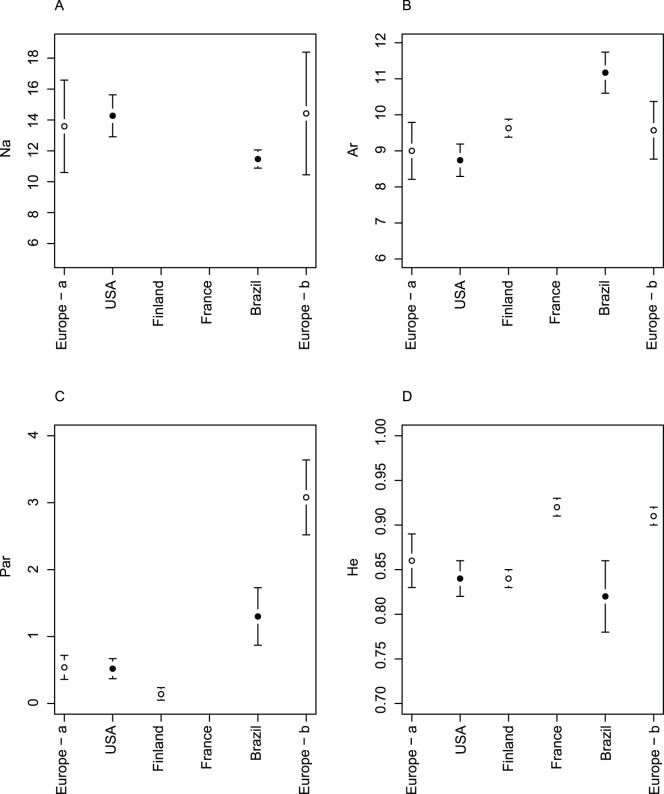
Comparison of different genetic diversity estimators: (Na) number of alleles (A); (Ar) allelic richness (B); (Par) private allelic richness (C); and (He) expected heterozygosity (D) from different house sparrow populations. For Europe –a and USA data from [24]; data for Finland from [58]; data for France from [59]; and data from Brazil and Europe –b where obtained from this study using all six loci (Table 1). Filled circles are introduced populations while open circles are native populations. Not all estimators were available in all the studies.

Brazilian house sparrow populations had significantly higher allelic richness (W = 23, p = 0.02) but significantly lower private allelic richness (W = 24, p<0.01), and lower unbiased He (W = 23, p = 0.01; see Table 1 for mean and sd) than European house sparrow populations. When only three loci were used to calculate allelic richness and private allelic richness, Brazilian populations were significantly lower (respectively: W = 24, p<0.01; W = 24, p = 0.01; see Table 1 for mean and sd). However, there was no significant difference in the mean number of alleles (W = 8.5, p = 0.52), observed heterozygosity (W = 6, p = 0.26) and *F_IS_* (W = 8, p = 0.48; see Table 1 for mean and sd) between introduced (Brazil) and native (Europe) house sparrow populations. However, when genetic diversity is compared with other house sparrow studies, populations from Brazil did not present lower genetic diversity; for allelic richness and private allelic richness they tended to present higher levels (Figure 2). Although we are unable to test statistically because of differences in microsatellites and number of loci used between the different studies, house sparrow populations in Brazil do not present a high loss of genetic variation. In support, none of the introduced populations from Brazil seemed to have experienced a significant bottleneck effect (lowest Wilcox one-tailed (heterozygosity excess) probability of 0.22). In the introduced range, there was no correlation between time since colonization and any of the genetic diversity indices (Na: r = −0.02, df = 4, p = 0.97; Ar: r = −0.01, df = 4, p = 0.99; Par: r = 0.24, df = 4, p = 0.64; Ho: r = 0.15, df = 4, p = 0.78). Altogether, our results do not support a scenario of sequential bottlenecks during the house sparrow expansion in Brazil or a major loss of genetic diversity.

### Population Genetic Differentiation

Genetic differentiation among European house sparrow populations was very low, both globally (*F_ST_* among European population  = 0.019; 95% C.I: 0.010–0.031) and in pair-wise comparisons (from 0.0043 to 0.0328; Table 3). However, all pairwise *F_ST_* values were significantly different for all European populations, except Italy and Spain. *D_est_* values for the different European populations were high (Table 3), suggesting that genetic differentiation is present in Europe.

**Table 3:** Pairwise *F_ST_* values for house sparrow populations in Europe (lower diagonal), values in bold are significantly different from zero after Bonferroni correction (p≤0.0083) and harmonic *D_est_* values (above the diagonal).

	Sweden	Bulgaria	Italy	Spain
Sweden	–	0.2000	0.2002	0.1210
Bulgaria	**0.0258**	–	0.2844	0.2491
Italy	**0.0219**	**0.0328**	–	0.0461
Spain	**0.0176**	**0.0262**	0.0043	–

For Brazil, genetic differentiation was also very low both globally (*F_ST_* among Brazilian population  = 0.028; 95% C.I: 0.016–0.046) and between population pairs (from 0.0050 to 0.0695; Table 4). However, two populations, Canoas and Niteroi, were significantly different from all other populations, and their pairwise *F_ST_* value was highest among all pairwise values (0.0695). Canoas is in the South of Brazil, while Niteroi is less than 10 km from Rio de Janeiro, where the house sparrows were initially released (Figure 1). Belém and Recife were also significantly differentiated. The pairwise *D_est_* values showed a similar pattern to the *F_ST_* values (Table 4), ranging from 0.0161 to 0.2510 and were highly correlated with *F_ST_* (Mantel r = 0.79, p = 0.013, 1000 randomisations); again, Niteroi and Canoas had the highest *D_est_* value. Furthermore, when *R_ST_* was used to infer genetic differentiation between house sparrow populations from Brazil, we found that global *R_ST_* values were higher than zero (*R_ST_* among Brazilian populations  = 0.033; 95% C.I.: 0.031–0.1111) and pairwise *R_ST_* values were similar to *F_ST_* and *D_est_* (Table 5) and highly correlated with *F_ST_* (Mantel r = 0.70, p = 0.035). The results from the *F_ST_*, *D_est_* and *R_ST_* analysis suggest slight genetic population differentiation in Brazil.

**Table 4:** Pairwise *F_ST_* values for house sparrow populations in Brazil (lower diagonal), values in bold are significantly different from zero after Bonferroni correction (p≤0.0033) and harmonic *D_est_* values (above the diagonal).

	Brasília	Cáceres	Belém	Recife	Niterói^1^	Canoas
Brasília	–	0.0580	0.1233	0.0558	0.1221	0.1646
Cáceres	0.0355	–	0.0161	0.0532	0.1888	0.0878
Belém	0.0235	0.0050	–	0.0803	0.1112	0.1382
Recife	0.0183	0.0098	**0.0128**	–	0.1130	0.1644
Niterói^1^	**0.0405**	**0.0313**	**0.0175**	**0.0278**	–	0.2510
Canoas	**0.0400**	**0.0316**	**0.0361**	**0.0268**	**0.0695**	–

1 - City closest to place of initial introduction.

**Table 5:** Pairwise *R_ST_* values for house sparrow populations in Brazil.

	Brasília	Cáceres	Belém	Recife	Niterói^1^	Canoas
Brasília	–					
Cáceres	−0.0073	–				
Belém	0.0107	0.0300	–			
Recife	−0.0046	−0.0063	0.0262	–		
Niterói^1^	0.0024	0.0490	0.0037	0.0373	–	
Canoas	0.0512	0.0326	0.0912	0.0407	0.1448	–

1 - City closest to place of initial introduction.

**Figure 3:**
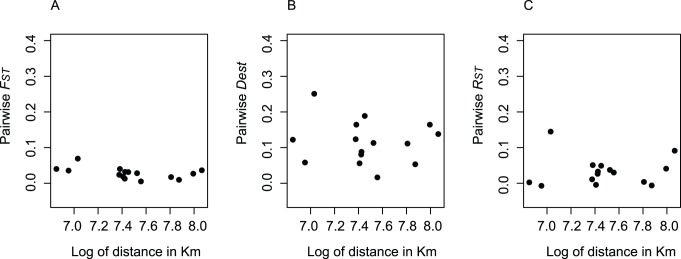
Scatterplots of *F_ST_* pairwise estimates [**62**] calculated using FSTAT version 2.9.3 [**54**] against geographical distance in km (log-transformed) for house sparrow populations of Brazil (A); pairwise harmonic mean *D_est_* [**63**] calculated using SMOGD [**64**] against geographic distance in km (log-transformed) for house sparrow populations of Brazil (B); and pairwise *R_ST_* calculated using R CALC [**66**] against geographic distance in km (log-transformed) for house sparrow populations of Brazil (C).

We found no isolation by distance, as shown by the non-significant negative correlation between genetic differentiation and geographic distance (*F_ST_*: Mantel r = −0.38, p = 0.13, 1000 randomisations (Figure 3A); *D_est_*: Mantel r = −0.10, p = 0.59, 1000 randomisations (Figure 3B); *R_ST_*: Mantel r = 0.03, p = 0.49 (Figure 3C)).

### Phenotypic Differentiation in Brazil

**Figure 4:**
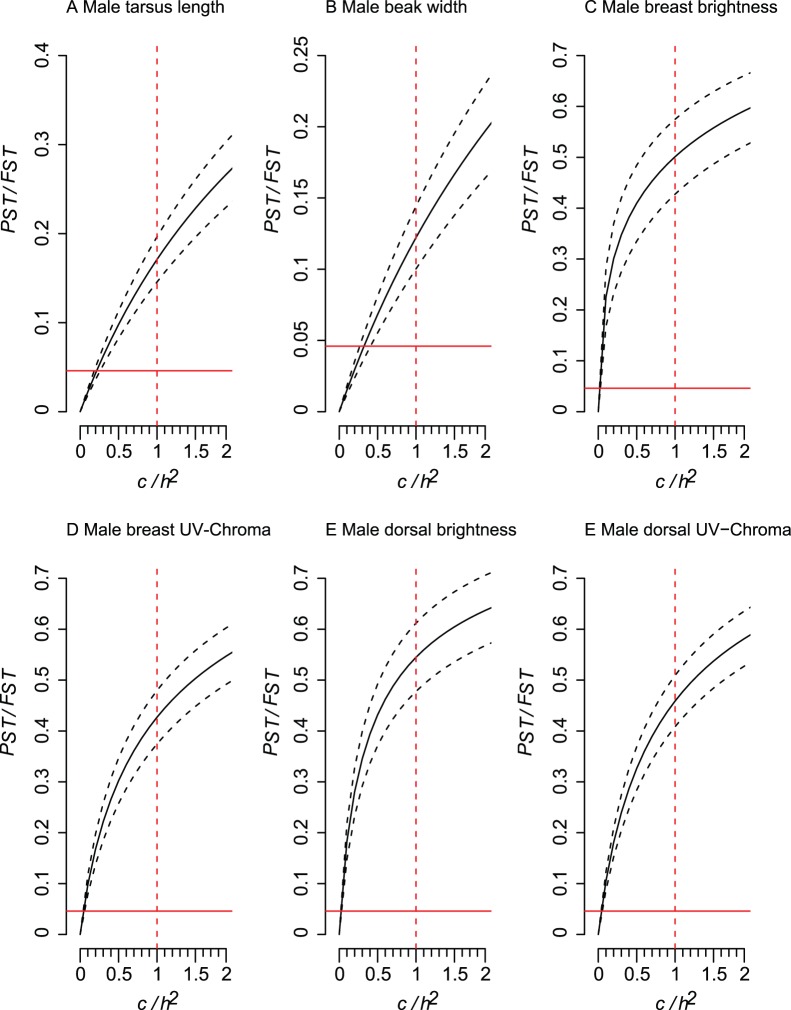
Comparison of phenotypic differentiation (*P_ST_* – solid line) with the upper 95% confidence interval (C.I.) for neutral genetic differentiation (*F_ST_*, solid red line), while the ratio *c/h^2^* was varied from zero to 2. The dashed black lines represents the 95% C.I. for the *P_ST_* calculations, while the dashed red line represent the null assumption that *c* = *h^2^*. Results are for male traits that had critical *c/h^2^* (the value in which the lower 95% C.I. of *P_ST_* is higher than the upper 95% C.I. of *F_ST_*) lower than 0.5. For values with higher critical value see Fig. S1.

**Figure 5:**
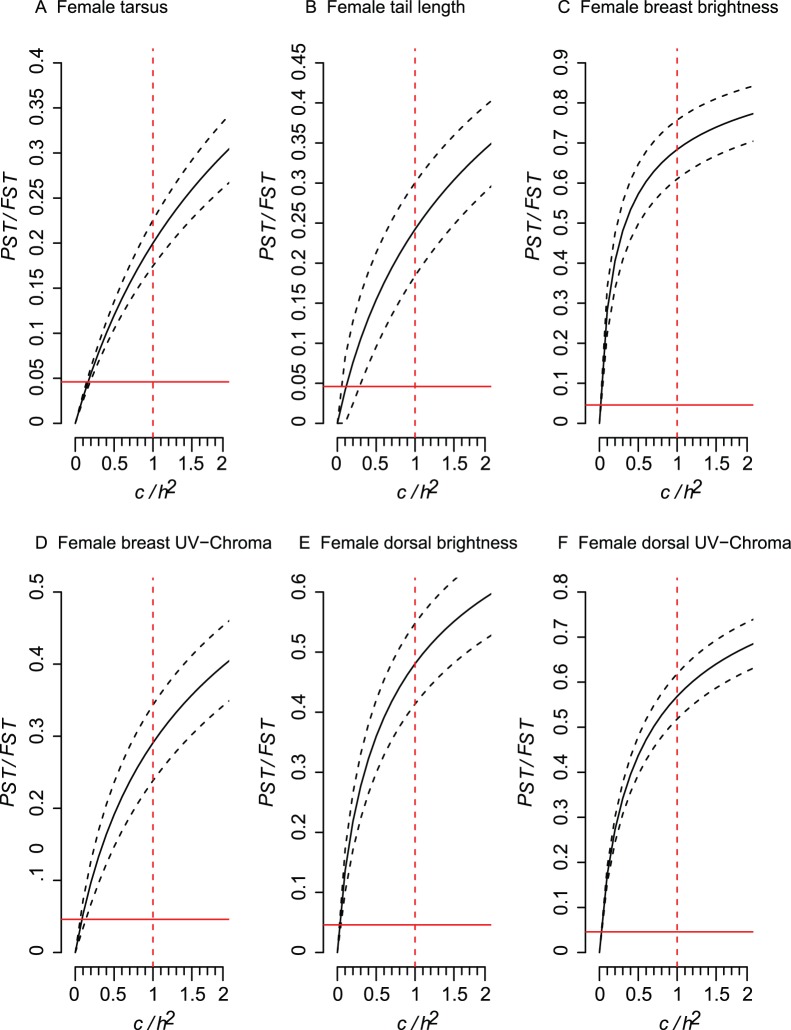
Comparison of phenotypic differentiation (*P_ST_* – solid line) with the upper 95% confidence interval (C.I.) for neutral genetic differentiation (*F_ST_*, solid red line), while the ratio *c/h^2^* was varied from zero to 2. The dashed black lines represents the 95% C.I. for the *P_ST_* calculations, while the dashed red line represent the null assumption that *c* = *h^2^*. Results are for female traits that had critical *c/h^2^* (the value in which the lower 95% C.I. of *P_ST_* is higher than the upper 95% C.I. of *F_ST_*) lower than 0.5. For values with higher critical value see Fig. S2.

Male and female morphologies differed, as shown by differences in population centroids (Female: *Pillai trace*  = 2.246, df = 55, 245 F = 3.633, p<0.001; Male: *Pillai trace*  = 1.897, df = 55, 370, F = 4.113, p<0.001). Comparisons of *P_ST_* with *F_ST_* show that similar traits in both males and females were usually shaped by selection (i.e., *P_ST_* higher than *F_ST_*) and not genetic drift, because lower 95% C.I. for *P_ST_* were higher than the upper 95% C.I. for *F_ST_* (Figure 4 and Figure 5). Evidence for the robustness of *P_ST_*>*F_ST_* varied among the traits but was exceptionally strong for plumage traits, which had critical *c/h^2^* lower than 0.10. Thus, the proportion of phenotypic variance across populations that is explained by additive genetic effects for plumage traits would need to be 10 times lower than the phenotypic variation encountered within populations for these traits to be explained by genetic drift. For tarsus length the additive genetic effects would need to be 5 times lower, while for wing length it would be less than two times. However, we also found similar traits in both males and females were *P_ST_* was either not higher than *F_ST_* or when higher critical *c/h^2^* was usually between 0.5 and 1.2 (Figure S1 and Figure S2), indicating that these traits are probably shaped by genetic drift. Therefore, phenotypic differentiation is low for these traits and the inference of selection acting on these traits is less robust than for the traits in Figure 4 and Figure 5.

## Discussion

The genetic variation in house sparrows from Brazil was only marginally lower compared to populations from the native range in Europe. We found no evidence for recent population bottlenecks or for the occurrence of sequential founder events during the range expansion process. We also found an absence of genetic structuring (or at most, weak structuring) among Brazilian populations, implying that expansion occurred with low influence of genetic drift and possibly high population growth. Moreover, we found that populations from Brazil differed morphologically from each other and that phenotypic divergence (*P_ST_*) was generally higher than expected from neutral genetic markers for similar traits in both males and females. However, our results must be interpreted with caution because of the small number of loci and populations used.

### Genetic Diversity

Private allelic richness (Par), and unbiased expected heterozygosity (UHe) were lower in introduced Brazilian than native European house sparrow populations. When only three loci were considered, both allelic richness (Ar) and Par were lower for introduced Brazilian populations. These results are consistent with founder effects observed with other bird introductions [20]–[23], [25]. However, observed heterozygosity (Ho), number of alleles (Na), and inbreeding (*F_IS_*) were not different from native European populations, and when six loci were used, we found higher Ar for introduced Brazilian house sparrow populations. Therefore, house sparrow populations in Brazil do not appear to have lost much genetic variation. In support, when genetic diversity estimators from this study were compared with the literature, we found that estimators were not substantially different from what is found in populations from the native range (Figure 2). Additionally, bottleneck signatures could not be detected for any Brazilian populations, although our small sample size may constrain our statistical power [60]. Moreover, time since colonization had no effect on genetic diversity, which suggests no occurrence of bottleneck or sequential founder events during the house sparrow expansion in Brazil. Thus, it seems that house sparrows in Brazil did not go through a strong population bottleneck and/or that once introduced to Brazil, population size quickly increased, thus reducing the effect of genetic drift [17], [30].

House sparrow studies from other introduced ranges have shown mixed results regarding the amount of genetic diversity lost when compared with the native range. For example, introduced populations in Australia and New Zealand exhibit a reduction in the number of alleles, but only the New Zealand populations had lower heterozygosity [26]. In North America, house sparrow populations had similar genetic diversity to native European populations, and in Kenya, introduced populations exhibit low levels of genetic diversity [24]. Differences in propagule pressure could explain the genetic diversity differences found in these distinctive introduced ranges. If so, introduced ranges derived from low propagule pressure should exhibit significant losses in genetic diversity, while introduced ranges from high propagule pressure should not present a reduction in genetic diversity [16], [18]. Although this pattern is maintained when we consider the North American introduction, with a release of over 1000 individuals over several events [3] with no reduction in genetic diversity, and the Australian and New Zealand introduction, which had over 300 individuals released over several events [3] and showed significant genetic losses, the same cannot be said about the Brazilian introduction. In Brazil, 100 pairs were introduced [44], therefore, significant losses in genetic diversity was expected, but substantial genetic loss was not found. It is possible that initial propagule pressure for Brazil was higher than indicated by historical records. Problems with the accuracy in historical records have been shown in the past [73] and care should be taken when using this kind of data to infer ecological processes. Another possibility is that rapid population growth could have occurred, which would have reduced the harmful effects associated with population bottleneck, allowing the retention of substantial genetic diversity [30], especially if consecutive bottlenecks or founder effects did not occur during the expansion [29]. Therefore, both differences in the introduction process and range expansions should influence genetic diversity.

### Population Genetic Differentiation in Brazil

We found low levels of genetic differentiation among house sparrow populations in Brazil. It seems that out of the six sampled populations only two, Canoas and Niteroi, are genetically different from all other populations. Canoas, which is in the south of Brazil (Figure 1), may be influenced by other house sparrow expansions. For example, 20 pairs were introduced in Buenos Aires, Argentina, in 1872 [3], and by 1888 house sparrows had already reached Uruguay [47], both of which border the south of Brazil. Possibly, house sparrow populations in the south of Brazil are an admixture of the two expanding populations, northward from Buenos Aires and southwards from Rio de Janeiro, which would explain why they are genetically different from the other populations in Brazil. However, Canoas presented the highest losses of genetic diversity (Table 1), which is not consistent with what would be expected for an admixed population. To test whether Canoas indeed has had a genetic influence from another expansion front, populations from both Uruguay and Argentina would have to be sampled.

Although Niteroi is very close to Rio de Janeiro, the cities are separated by Guanabara Bay, a large body of water that the house sparrows would need to cross, or alternatively take a longer inland route between the two cities. Perhaps the population of Niteroi had a higher influence of genetic drift when compared to other populations, which could be the case if colonization of Niteroi occurred before house sparrows could reach it via an inland route. Thus, Niteroi may not provide a good representation of the initial founding population of Rio de Janeiro. We also found no isolation by distance for populations, and *F_ST_* variation was not high, suggesting that the influence of genetic drift was low [5], [31]. These results indicate that: (1) the source population was genetically homogeneous prior to the introduction (i.e., consistent with one introductory event); and (2) the expansion process probably occurred with high population growth and large propagule size from within the introduced range, which reduced the effects of genetic drift. In other introduced ranges, house sparrow populations also present weak genetic differentiation [24], [26], with the exception of Australia, in which populations are significantly more differentiated compared to New Zealand and Britain (most likely ancestral source population). Therefore, the low influence of genetic drift seems to be a common feature in the expansion process of house sparrows in introduced ranges. Moreover, house sparrows in the native range also present low levels of genetic differentiation [24], [58], [59] and it is possible that the evolutionary history of house sparrows in the native range may have influenced the genetic diversity captured during invasion [74], i.e, house sparrow populations from the introduced range are simply reflecting the geographical genetic structure of the native range. For example, the lack of genetic structure in the native range may result in low levels of population admixture in the introduced range.

A possible explanation for the low influence of genetic drift in house sparrow introduced ranges could be that there has not been enough time for genetic drift to take place because most introductions occurred around 1850 [3]. However, house sparrows are sedentary birds in their native range with natal dispersal distance of about 2 km [40], and populations in the native range also present low genetic differentiation [24], [58], [59]. Therefore, gene flow may be comparatively high in this species. Data on dispersal distances in introduced ranges are available for North America, which show similarly short dispersal distances [40]. If dispersal distance in the other introduced ranges is similar to that in the native range, which might be the case, and if colonization distance is also associated with dispersal distance, then it is possible that the expansion of house sparrows in the introduced ranges (Brazil, North America and New Zealand) has been a contiguous process with high gene flow among the new founding populations with high population growth.

### Morphological Differentiation in Brazil

Morphological divergence was found among house sparrow populations in Brazil and *P_ST_*- *F_ST_* analysis indicates that divergence of most of the morphological traits was due to selection and not genetic drift, with the exception of wing length and most beak measurements.

House sparrow populations from other introduced ranges, such as North America [34], New Zealand [42] and Hawaii [75], have also shown substantial morphological divergence. The latter study has also shown that morphological divergence was mainly due to selection and not genetic drift. Influence of genetic drift is higher in small populations [30], but because house sparrows probably quickly expanded in the introduced ranges and, therefore, had large populations sizes, it can be expected that genetic drift did not play a substantial role in the shaping of most morphological traits.

Although divergent selection (favouring of different phenotypes in different populations) may be driving morphological divergence of some of the traits, it is difficult to discern whether this pattern is a response to selection (microevolution) or simply a plastic response to the environment. In this study it is more challenging to evaluate this because we used *P_ST_* instead of the more accurate *Q_ST_* [39]_._ Our *P_ST_* estimates therefore cannot rule out environmental or parental (e.g. differences in parental care) effects on morphological traits. Nonetheless, without genetic differences between the introduced populations, it seems more plausible that phenotypic plasticity is driving morphological differentiation in some of the traits. However, if selection is indeed responsible for phenotypic divergence, one can expect it to occur in a predictable manner, such as local adaptation to the abiotic environment [37]. It has already been shown that this could be the case for house sparrow populations of North America, where a positive correlation was found between body size and latitude [34], [41]. However, no correlation was found between house sparrow morphological traits with latitude in South America [41], which could be indicative of a lack of local adaptation and that genetic drift may be driving morphological divergence in South American populations. Our data show that traits related to body size, such as wing length, tarsus length and tail length did not present a robust critical *c/h^2^* when compared to other traits such as plumage coloration. Therefore, phenotypic differentiation of these traits may not have a very strong adaptive basis for house sparrow populations in Brazil. A similar result was also found for native house sparrow populations from Finland, where only body mass across populations seemed to be adaptive, while other traits (bill, wing and tarsus length) seemed to be shaped by genetic drift [71].

However, we must interpret our results cautiously because of the small number of populations used in our *P_ST_*−*F_ST_* analysis. Also, because two of the microsatellites had a high number of alleles, and therefore high-expected heterozygosity that can generate low levels of *F_ST_*, it is possible that type-I errors of rejecting the null hypothesis *P_ST_* = *F_ST_* may have occurred [9]. However, our study is the first step in understanding the adaptive potential of invasive populations of house sparrows in Brazil, and our initial data show that we may expect to find high plumage differentiation among populations of house sparrows in Brazil. Therefore, future efforts should explore why plumage may have a higher phenotypic differentiation when compared to other phenotypic traits.

### Conclusions

Introduced house sparrow populations from Brazil lost some genetic variation relative to sparrows from the native range in Europe. However, it seems that the expansion process occurred in association with high population growth and possibly gene flow, thus enabling populations from Brazil to retain substantial genetic diversity with little genetic differentiation. However, our results need to be interpreted cautiously because of the low number of markers and populations used. We found significant morphological variation among populations and, overall, morphological divergence was higher than neutral genetic divergence suggesting the action of selection overriding the effect of genetic drift for many of the traits when *F_ST_* was used. However, not all the traits presented *P_ST_*>*F_ST_* and traits related to body size (tarsus, wing and tail length) were less robust in the sensitive analysis then plumage traits. Using the *P_ST_*−*F_ST_* approach as an initial step allows us to infer that house sparrows should quickly respond to new selective factors they are exposed to in new areas, especially to factors affecting plumage coloration. In addition, future experimental studies should be able to determine if the morphological divergence observed in Brazil is due to microevolution (changes in genotype frequency) or plastic phenotypic responses to environmental conditions.

## Supporting Information

Figure S1 **Comparison of phenotypic differentiation (** ***P_ST_*** **– solid line) with the upper 95% confidence interval (C.I.) for neutral genetic differentiation (** ***F_ST_*** **, solid red line), while the ratio** ***c/h^2^*** **was varied from zero to 2.** The dashed black lines the 95% C.I. for the *P_ST_* calculations, while the dashed red line represent the null assumption that *c* = *h^2^*. Results are for male traits that had critical *c/h^2^* (the value in which the lower 95% C.I. of *P_ST_* is higher than the upper 95% C.I. of *F_ST_*) higher than 0.6.
(EPS)

Figure S2 **Comparison of phenotypic differentiation (** ***P_ST_*** **– solid line) with the upper 95% confidence interval (C.I.) for neutral genetic differentiation (** ***F_ST_*** **, solid red line), while the ratio** ***c/h^2^*** **was varied from zero to 2.** The dashed black lines represents the 95% C.I. for the *P_ST_* calculations, while the dashed red line represent the null assumption that *c* = *h^2^*. Results are for female traits that had critical *c/h^2^* (the value in which the lower 95% C.I. of *P_ST_* is higher than the upper 95% C.I. of *F_ST_*) higher than 0.5.
(EPS)

Information S1 **PCR Cycling conditions.**
(DOCX)

## References

[1] Sax DF, Stachowicz JJ, Brown JH, Bruno JF, Dawson MN, et al (2007) Ecological and evolutionary insights from species invasions. Trends Ecol Evol 22: 465–471 doi:10.1016/j.tree.2007.06.009. 17640765 10.1016/j.tree.2007.06.009

[2] Duncan R, Blackburn T, Sol D (2003) The ecology of bird introductions. Ann Rev Ecol Evol Syst 34: 71–98 doi:10.1146/annurev.ecolsys.34.011802.132353.

[3] Long JL (1981) Introduced Birds of the World. Hong Kong: A. H. & A. W. Reed PTY Ltd. 528 p.

[4] Lee CE (2002) Evolutionary genetics of invasive species. Trends Ecol Evol 17: 386–391.

[5] Ramakrishnan AP, Musial T, Cruzan MB (2010) Shifting dispersal modes at an expanding species’ range margin. Mol Ecol 19: 1134–1146 doi:10.1111/j.1365-294X.2010.04543.x. 20456225 10.1111/j.1365-294X.2010.04543.x

[6] Uller T, Leimu R (2011) Founder events predict changes in genetic diversity during human-mediated range expansions. Global Change Biol 17: 3478–3485 doi:10.1111/j.1365-2486.2011.02509.x.

[7] Baker AJ (1992) Genetic and morphometric divergence in ancestral European and descendent New Zealand populations of chaffinches (*Fringilla coelebs*). Evolution 46: 1784–1800. 28567765 10.1111/j.1558-5646.1992.tb01169.x

[8] Bossdorf O, Auge H, Lafuna L, Rogers WE, Siemann E, et al (2005) Phenotypic and genetic differentiation between native and introduced plant populations. Oecologia 144: 1–11 doi:10.2307/20062297. 15891837 10.1007/s00442-005-0070-z

[9] Keller SR, Taylor DR (2008) History, chance and adaptation during biological invasion: separating stochastic phenotypic evolution from response to selection. Ecol Lett 11: 852–866 doi:10.1111/j.1461-0248.2008.01188.x. 18422638 10.1111/j.1461-0248.2008.01188.x

[10] Huey RB, Gilchrist GW, Hendry AP (2005) Using invasive species to study evolution: cases studies with *Drosophila* and salmon In: Sax DF, Stachowicz JJ, Gaines ST, editors. Species Invasions: Insights into Ecology, Evolution and Biogeography. Sunderland: Sinauer Associates, Inc. 139–164.

[11] Kolar CS, Lodge DM (2001) Progress in invasion biology: predicting invaders. Trends Ecol Evol 16: 199–204. 11245943 10.1016/s0169-5347(01)02101-2

[12] Sakai AK, Allendorf FW, Holt JS, Lodge DM, Molofsky J, et al (2001) The population biology of invasive species. Annu Rev Ecol Syst 32: 305–332.

[13] Holt RD, Barfield M, Gomulkiewicz R (2005) Theories of niche conservatism and evolution: could exotic species be potential tests. In: Sax DF, Stachowicz JJ, Gaines MS, editors. Species Invasion: Insights into Ecology, Evolution, and Biogeography. Sunderland: Sinauer Associates, Inc. 259–290.

[14] Suarez AV, Tsutsui ND (2008) The evolutionary consequences of biological invasions. Mol Ecol 17: 351–360 doi:10.1111/j.1365-294X.2007.03456.x. 18173507 10.1111/j.1365-294X.2007.03456.x

[15] Simberloff D (2009) The role of propagule pressure in biological invasions. Ann Rev Ecol Evol Syst 40: 81–102 doi: 10.1146/annurev.ecolsys.110308.120304.

[16] Allendorf F, Lundquist L (2003) Introduction: population biology, evolution, and control of invasive species. Conserv Biol 17: 24–30.

[17] Wares JP, Hughes AR, Grosberg RK (2005) Mechanisms that drive evolutionary change: insights from species introductions and invasions. In: Sax DF, Stachowicz JJ, Gaines MS, editors. Species Invasions: Insights into Ecology, Evolution and Biogeography. Sunderland: Sinauer Associates, Inc. 229–257.

[18] Dlugosch KM, Parker IM (2008) Founding events in species invasions: genetic variation, adaptive evolution, and the role of multiple introductions. Mol Ecol 17: 431–449 doi:10.1111/j.1365-294X.2007.03538.x. 17908213 10.1111/j.1365-294X.2007.03538.x

[19] Clegg SM, Degnan SM, Kikkawa J, Moritz C, Estoup A, et al (2002) Genetic consequences of sequential founder events by an island-colonizing bird. Proc Natl Acad Sci U S A 99: 8127–8132. 12034870 10.1073/pnas.102583399 PMC123032

[20] Baker AJ, Moeed A (1987) Rapid genetic differentiation and founder effect in colonizing populations of common mynas (*Acridotheres tristis*). Evolution 41: 525–538. 28563814 10.1111/j.1558-5646.1987.tb05823.x

[21] Cabe PR (1998) The effects of founding bottlenecks on genetic variation in the European starling (*Sturnus vulgaris*) in North America. Heredity 80: 519–525.

[22] Hawley D, Hanley D, Dhondt A, Lovette IJ (2006) Molecular evidence for a founder effect in invasive house finch (*Carpodacus mexicanus*) populations experiencing an emergent disease epidemic. Mol Ecol 15: 263–275 doi:10.1111/j.1365-294X.2005.02767.x. 16367845 10.1111/j.1365-294X.2005.02767.x

[23] Merilä J, Björklund M, Baker AJ (1996) The successful founder: genetics of introduced *Carduelis chloris* (greenfinch) populations in New Zealand. Heredity 77: 410–422 doi:10.1038/hdy.1996.161.

[24] Schrey A, Grispo M, Awad M, Cook M, McCoy ED, et al (2011) Broad-scale latitudinal patterns of genetic diversity among native European and introduced house sparrow (*Passer domesticus*) populations. Mol Ecol 20: 1133–1143 doi:10.1111/j.1365-294X.2011.05001.x. 21251113 10.1111/j.1365-294X.2011.05001.x

[25] St Louis VL, Barlow JC (1988) Genetic differentiation among ancestral and introduced populations of the Eurasian tree sparrow (*Passer montanus*). Evolution 42: 266–276. 28567836 10.1111/j.1558-5646.1988.tb04131.x

[26] Parkin DT, Cole SR (1985) Genetic differentiation and rates of evolution in some introduced populations of the house sparrow, *Passer domesticus* in Australia and New Zealand. Heredity 54: 15–23.

[27] Ross HA (1983) Genetic differentiation of starling (Sturnus vulgaris: Aves) populations in New Zealand and Great Britain. Journal of Zoology 201: 351–362.

[28] Blackburn TM, Lockwood JL, Cassey P (2009) Avian Invasions: the Ecology and Evolution of Exotic Birds?. Oxford: Oxford University Press. 305 p.

[29] Excoffier L, Foll M, Petit RJ (2009) Genetic consequences of range expansions. Ann Rev Ecol Evol Syst 40: 481–501 doi:10.1146/annurev.ecolsys.39.110707.173414.

[30] Nei M, Maruyama T, Chakraborty R (1975) The bottleneck effect and genetic variability in populations. Evolution 29: 1–10. 28563291 10.1111/j.1558-5646.1975.tb00807.x

[31] Hutchison DW, Templeton AR (1999) Correlation of pairwise genetic and geographic distance measures: inferring the relative influences of gene flow and drift on the distribution of genetic variability. Evolution 53: 1898–1914. 28565459 10.1111/j.1558-5646.1999.tb04571.x

[32] Broennimann O, Treier UA, Müller-Scharer H, Thuiller W, Peterson AT, et al (2007) Evidence of climatic niche shift during biological invasion. Ecol Lett 10: 701–709. 17594425 10.1111/j.1461-0248.2007.01060.x

[33] Huey RB, Gilchrist GW, Carlson ML, Berrigan D, Serra L (2000) Rapid evolution of a geographic cline in size in an introduced fly. Science 287: 308–309. 10634786 10.1126/science.287.5451.308

[34] Johnston RF, Selander RK (1971) Evolution in the house sparrow. II. Adaptive differentiation in North American populations. Evolution 25: 1–28. 28562938 10.1111/j.1558-5646.1971.tb01855.x

[35] Leinonen T, O'Hara RB, Cano JM, Merilä J (2008) Comparative studies of quantitative trait and neutral marker divergence: a meta-analysis. J Evol Biol 21: 1–17 doi:10.1111/j.1420-9101.2007.01445.x. 18028355 10.1111/j.1420-9101.2007.01445.x

[36] Merilä J, Crnokrak P (2001) Comparison of genetic differentiation at marker loci and quantitative traits. J Evol Biol 14: 892–903.

[37] Whitlock MC (2008) Evolutionary inference from QST. Mol Ecol 17: 1885–1896 doi:10.1111/j.1365-294X.2008.03712.x. 18363667 10.1111/j.1365-294X.2008.03712.x

[38] Spitze K (1993) Population structure in *Daphnia obtusa*: quantitative genetic and allozymic variation. Genetics 135: 367–374. 8244001 10.1093/genetics/135.2.367 PMC1205642

[39] Pujol B, Wilson AJ, Ross RIC, Pannell JR (2008) Are QST-FST comparisons for natural populations meaningful? Mol Ecol 17: 4782–4785 doi:10.1111/j.1365-294X.2008.03958.x. 19140971 10.1111/j.1365-294X.2008.03958.x

[40] Anderson T (2006) Biology of the Ubiquitous House Sparrow: from Genes to Populations. New York: Oxford University Press. 547 p.

[41] Johnston RF, Selander RK (1973) Evolution in the house sparrow. III. Variation in size and sexual dimorphism in Europe and North and South America. Am Nat 107: 373–390.

[42] Baker A (1980) Morphometric differentiation in New Zealand populations of the house sparrow (*Passer domesticus*). Evolution 34: 638–653. 28563981 10.1111/j.1558-5646.1980.tb04003.x

[43] Marzal A, Ricklefs RE, Valkiūnas G, Albayrak T, Arriero E, et al (2011) Diversity, loss, and gain of malaria parasites in a globally invasive bird. PLoS ONE 6: e21905 doi:10.1371/journal.pone.0021905.t001. 21779353 10.1371/journal.pone.0021905 PMC3136938

[44] Sick H (1997) Ornitologia brasileira. Rio de Janeiro: Editora Nova Fronteira. 827 p.

[45] Borges SH, Pacheco JF, Whittaker A (1996) New records of the house sparrow (*Passer domesticus*) in the Brazilian Amazon. Ararajuba 4: 116–117.

[46] Silva J, Oren D (1990) Introduced and invading birds in Belém, Brazil. Wilson Bull 102: 309–313.

[47] Sick H (1959) Invasão da América Latina pelo pardal, *Passer domesticus* Linnaeus 1758, com referência especial ao Brasil. B Mus Nac 207: 1–31.

[48] Smith NJH (1973) House sparrows (*Passer domesticus*) in the Amazon. Condor 75: 242–243.

[49] Smith NJH (1980) Further advances of house sparrows into the Brazilian Amazon. Condor 82: 109–111.

[50] Sambrook J, Russel D (2001) Molecular Cloning: a Laboratory Manual. Cold Spring Harbor, New York: Cold Spring Harbor Laboratory Press. 2344 p.

[51] Dawson D, Burke T, Hansson B, Pandhal J, Hale MC, et al (2006) A predicted microsatellite map of the passerine genome based on chicken-passerine sequence similarity. Mol Ecol 15: 12991320. 10.1111/j.1365-294X.2006.02803.x 16626455

[52] Griffith SC, Stewart IRK, Dawson DA, Owens IPF, Burke T (1999) Contrasting levels of extra-pair paternity in mainland and island populations of the house sparrow (*Passer domesticus*): is there an 'island effect'? Biol J Linn Soc Lond 68: 303–316.

[53] Neumann K, Wetton JH (1996) Highly polymorphic microsatellites in the house sparrow *Passer domesticus* . Mol Ecol 5: 307–309. 8673277

[54] Goudet J (1995) FSTAT (Version 1.2): A computer program to calculate F-statistics. J Hered 86: 485–486.

[55] Van Oosterhout C, Hutchinson W, Wills D, Shipley P (2004) MICRO-CHECKER: software for identifying and correcting genotyping errors in microsatellite data. Mol Ecol Notes 4: 535–538 doi:10.1111/j.1471-8286.2004.00684.x.

[56] Kalinowski ST (2004) Counting alleles with rarefaction: private alleles and hierarchical sampling designs. Conserv Genet 5: 539–543.

[57] Peakall R, Smouse PE (2006) GENALEX 6: genetic analysis in Excel. Population genetic software for teaching and research. Mol Ecol Notes 6: 288–295 doi:10.1111/j.1471-8286.2005.01155.x. 10.1093/bioinformatics/bts460 PMC3463245 22820204

[58] Kekkonen J, Seppä P, Hanski IK, Jensen H, Väisänen RA, et al (2010) Low genetic differentiation in a sedentary bird: house sparrow population genetics in a contiguous landscape. Heredity 106: 183–190 doi:10.1038/hdy.2010.32. 20372181 10.1038/hdy.2010.32 PMC3183854

[59] Loiseau C, Richard M, Garnier S, Chastel O, Julliard R, et al (2009) Diversifying selection on MHC class I in the house sparrow (*Passer domesticus*). Mol Ecol 18: 1331–1340 doi:10.1111/j.1365-294X.2009.04105.x. 19368641 10.1111/j.1365-294X.2009.04105.x

[60] Cornuet JM, Luikart G (1996) Description and power analysis of two tests for detecting recent population bottlenecks from allele frequency data. Genetics 144: 2001–2014. 8978083 10.1093/genetics/144.4.2001 PMC1207747

[61] Piry S, Luikart G, Cournet JM (1999) BOTTLENECK: a computer program for detecting recent reductions in the effective size using allele frequency data. J Hered 90: 502–503 doi:10.1093/jhered/90.4.502.

[62] Weir BS, Cockerham CC (1984) Estimating F-Statistics for the analysis of population structure. Evolution 38: 1358–1370. 28563791 10.1111/j.1558-5646.1984.tb05657.x

[63] Jost L (2008) GST and its relatives do not measure differentiation. Mol Ecol 17: 4015–4026 doi:10.1111/j.1365-294X.2008.03887.x. 19238703 10.1111/j.1365-294x.2008.03887.x

[64] Crawford N (2010) SMOGD: software for the measurement of genetic diversity. Mol Ecol Resour 10: 556–557 doi:10.1111/j.1755-0998.2009.02801.x. 21565057 10.1111/j.1755-0998.2009.02801.x

[65] Slatkin M (1995) A measure of population subdivision based on microsatellite allele frequencies. Genetics 139: 457–462. 7705646 10.1093/genetics/139.1.457 PMC1206343

[66] Goodman S (1997) RST Calc: a collection of computer programs for calculating estimates of genetic differentiation from microsatellite data and determining their significance. Mol Ecol 6: 881–885.

[67] Excoffier L, Laval G, Schneider S (2005) Arlequin (version 3.0): An integrated software package for population genetics data analysis. Evol Bioinform 1: 47–50. PMC2658868 19325852

[68] Oksanen J, Blanchet FG, Kindt R, Sólymos P, Stevens MHH, et al (2011) Vegan: community ecology package. R package version 20–2: 1–43.

[69] Jensen H, Saether B-E, Ringsby TH, Tufto J, Griffith SC, et al (2003) Sexual variation in heritability and genetic correlations of morphological traits in house sparrow (*Passer domesticus*). J Evol Biol 16: 1296–1307 doi:10.1046/j.1420-9101.2003.00614.x. 14640421 10.1046/j.1420-9101.2003.00614.x

[70] Brommer JE (2011) Whither Pst? The approximation of Qst by Pst in evolutionary and conservation biology. J Evol Biol 24: 1160–1168 doi: 10.1111/j.1420-9101.2011.02268.x. 21457173 10.1111/j.1420-9101.2011.02268.x

[71] Kekkonen J, Jensen H, Brommer JE (2012) Morphometric differentiation across House Sparrow *Passer domestic*us populations in Finland in comparison with the neutral expectation for divergence. Ibis 154: 846–857 doi: 10.1111/j.1474-919X.2012.01252.x.

[72] Filzmoser P, Gschwandtner M (2011) mvoutlier: multivariate outlier detection based on robust methods. R package version 194.

[73] Moulton MP, Cropper WP, Avery ML, Moulton LE (2010) The earliest house sparrow introductions to North America. Biol Invasions 12: 2955–2958 doi: 10.1007/s10530-010-9692-0.

[74] Taylor DR, Keller SR (2007) Historical range expansion determines the phylogenetic diversity introduced during contemporary species invasions. Evolution 61: 334–345 doi:10.1111/j.1558-5646.2007.00037.x. 17348944 10.1111/j.1558-5646.2007.00037.x

[75] Mathys BA, Lockwood JL (2011) Contemporary morphological diversification of passerine birds introduced to the Hawaiian archipelago. Proc R Soc B: Biol Sci 278: 2392–2400 doi:10.1098/rspb.2010.2302. 10.1098/rspb.2010.2302 PMC3119009 21208954

