# Supplementary material for: Genetic and Morphometric Divergence of an Invasive Bird: The Introduced House Sparrow (Passer domesticus) in Brazil
Source: PLoS One. 2012 Dec 28;7(12):e53332. doi: 10.1371/journal.pone.0053332 (PMC3532305; doi:10.1371/journal.pone.0053332)
Supplement: Information S1 — PCR Cycling conditions. (DOCX) [file pone.0053332.s003.docx]

**Supplementary Methods**

**PCR Cycling conditions:**

*Pdoµ1:*

95ºC denaturing step for 15 min, followed by 28 cycles of 95ºC for 30 s, primer specific annealing temperature of 64ºC for 1 min, 72ºC for 1 min and a final elongation step of 5 min at 60ºC.

*Pdoµ3:*

We used a Touch Down (TD) PCR procedure followed by normal PCR cycling.

TD conditions: 95ºC denaturing step for 15 min, followed by 12 cycles of 95ºC for 1min, primer specific annealing temperature of 69ºC for one 1 min (minus 1 ºC in the subsequent 12 cycles) and 72ºC elongation step for 1 min; this was subsequently followed by 20 cycles of 95ºC for 30s, primer annealing temperature of 57ºC for 1 min, 72ºC for 1 min and a final elongation step of 5 min at 60ºC.

*Pdoµ4:*

95ºC denaturing step for 15 min, followed by 35 cycles of 95ºC for 30 s, primer specific annealing temperature of 55ºC for 1 min, 72ºC for 1 min and a final elongation step of 5 min at 60ºC.

*Pdoµ6:*

95ºC denaturing step for 15 min, followed by 28 cycles of 95ºC for 30 s, primer specific annealing temperature of 64ºC for 1 min, 72ºC for 1 min and a final elongation step of 5 min at 60ºC.

*Pdo8:*

TD conditions: 95ºC denaturing step for 15 min, followed by 12 cycles of 95ºC for 1min, primer specific annealing temperature of 70ºC for one 1 min (minus 1 ºC in the subsequent 12 cycles) and 72ºC elongation step for 1 min; this was subsequently followed by 20 cycles of 95ºC for 30s, primer annealing temperature of 59ºC for 1 min, 72ºC for 1 min and a final elongation step of 5 min at 60ºC.

*Pdo9:*

TD conditions: 95ºC denaturing step for 15 min, followed by 12 cycles of 95ºC for 1min, primer specific annealing temperature of 60ºC for one 1 min (minus 1 ºC in the subsequent 12 cycles) and 72ºC elongation step for 1 min; this was subsequently followed by 20 cycles of 95ºC for 30s, primer annealing temperature of 49ºC for 1 min and 30s, 72ºC for 1 min and a final elongation step of 5 min at 60ºC.
